# 

**Published:** 1883-11

**Authors:** 


					﻿NOVEMBER, 1883.
THIRTIETH YEAR.
HALL’S
Ji OUÏBÎÏÆJEi
OF
HEALTH
Guaranteed Circulation 200,000 Copies in 12 Months.
E. H. GIBBS, A.M., M.D., Editor,
No. 21 CLINTON PLACE, EIGHTH STEEET, NEW YORK.
SAMPLE COPY.
I TERMS: $1 a Year.
SiDgle nnmlier, 10 cts.
				

